# Clinical and cytogenetic characteristics of choroidal melanoma in Vietnamese Asians

**Published:** 2011-01-21

**Authors:** Tara A. McCannel, Melinda Y. Wu, Barry L. Burgess

**Affiliations:** 1 Department of Ophthalmology and the Jules Stein Eye Institute, University of California, Los Angeles, Los Angeles, CA; 2 Washington University School of Medicine, St. Louis, MO

## Abstract

**Purpose:**

To report the clinical and cytogenetic characteristics of choroidal melanoma in Vietnamese Asians.

**Methods:**

In three Vietnamese Asians with choroidal melanoma, transscleral fine needle aspiration biopsy (FNAB) was performed immediately before Iodine-125 brachytherapy. Biopsy was examined for cytopathology, fluorescence in situ hybridization (FISH) for the centromere of chromosome 3, and analyzed by 250K whole genome Mapping Array and U133 plus 2.0 Expression Array.

**Results:**

Three Vietnamese Asian men (50, 59, and 30 years of age) with clinical diagnosis of choroidal melanoma and no evidence of metastasis had FNAB immediately before Iodine-125 brachytherapy. Cytopathology showed heavily pigmented cells suggestive of or consistent with melanoma. Mapping Array and Expression Array revealed cytogenetic aberrations and gene expression profiles characteristic of choroidal melanoma. One patient (Case 2) with chromosome 3 loss and chromosome 8q gain developed biopsy-proven liver metastasis three years after brachytherapy. One patient (Case 1) with chromosome 6p, 9q and 17q gain and a second patient (Case 3) with 6p, 8q and 9q gains and losses in 6q and 8p have had no evidence of metastasis three years after brachytherapy.

**Conclusions:**

In this series of Vietnamese Asians with heavily pigmented choroidal melanoma, the clinical characteristics, cytogenetic aberrations and gene expression profiles were similar to characteristics in other ethnic/racial groups and the cytogenetic aberration of chromosome 3 loss was associated with the development of liver metastasis.

## Introduction

Choroidal melanoma is the most common primary intraocular malignancy in adults. Studies from various countries have reported annual incidence rates between 5.3 and 10.9 cases per million [1]. A recent report found that the annual incidence of choroidal melanoma in the United States per million was 6.02 in non-Hispanic whites, 1.67 in Hispanics, 0.38 in Asians, 0 in Native Americans, and 0.31 in blacks [2].

Asian refers to persons with origin in any of the original peoples of Asia and is further characterized by culture and ancestry to a specific country or region [3]. In this context, choroidal melanoma has been reported in Japanese Asians, Chinese Asians, Thai Asians, and others of Asian origin [4-7]. We are not aware, however, of a prior report of choroidal melanoma in Vietnamese Asians.

We report a series of three Vietnamese Asians with choroidal melanoma. The clinical presentation and clinical course of disease, the cytogenetic aberrations and the gene expression profiles revealed by high-density single nucleotide polymorphism (SNP) mapping array and expression array are presented.

## Methods

All studies were performed in accordance with the Declaration of Helsinki and the United States Health Insurance Portability and Accountability Act (HIPAA) of 1996. All participants gave informed consent and all studies were approved by the Office of Human Research Protection (Institutional Review Board) of the University of California, Los Angeles.

Patients were self-declared Vietnamese Asians on the basis of Vietnamese ancestry and identification with the social and cultural characteristics of Vietnam [3].

Clinical diagnosis of choroidal melanoma was established by comprehensive ophthalmology examination, fundus photography, fluorescein angiography, and ultrasonography.

Transscleral fine needle aspiration biopsy (FNAB), cytopathology, fluorescence in situ hybridization (FISH) for the centromere of chromosome 3, and analysis by 250K Mapping Array (Affymetrix, Santa Clara, CA) for chromosome copy number and U133 plus 2.0 Array (Affymetrix) for gene expression were performed as described previously  [8-11]. Briefly, probe preparation, hybridization, and reading were performed by the UCLA DNA Microarray Core (Los Angeles, CA), according to the standard 96-well protocol published by Affymetrix. Copy number variation was computed using CNAT v4.0.1 software from Affymetrix.

## Results

### Case 1

**Figure 1:**
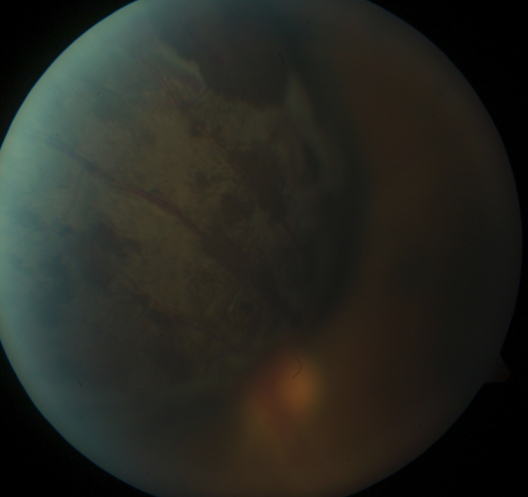
Color fundus photograph of the left eye in Case 1 at initial presentation demonstrates a superonasal pigmented choroidal melanoma.

A previously healthy 50-year-old Vietnamese man presented with a three month history of photopsia and “black vision” in the left eye. Visual acuity was 20/20 in the right eye and Count Fingers at 3 feet in the left eye. The left eye had an inferior and central field defect and left exotropia was present. The right eye was unremarkable. Funduscopy of the left eye revealed a heavily pigmented choroidal tumor in the superonasal periphery with extensive subretinal hemorrhage in the superior fundus and upper part of the macula (Figure 1). Ultrasonography showed a mushroom-shaped choroidal tumor with low to medium reflectivity, height of 8.10 mm and basal dimensions of 9.99 mm by 11.20 mm.

**Figure 2:**
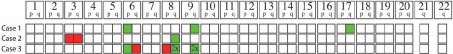
Chromosomal aberrations determined by high-density genome single nucleotide polymorphism mapping (SNP) array in fine needle aspiration biopsy specimens. Summary of accumulated chromosomal gain and loss data from GeneChip 250k NspI mapping arrays for biopsies of the three patients is demonstrated. Red boxes denote whole or partial loss; green boxes denote a whole or partial gain; green boxes labeled 2× denote a two-copy gain.

With clinical diagnosis of choroidal melanoma, left eye, and no evidence of metastasis, the patient was treated with Iodine-125 brachytherapy and transscleral fine needle aspiration biopsy (FNAB) immediately before plaque placement. Cytopathology showed pigmented cells suspicious for melanoma and fluorescence in situ hybridization (FISH) for chromosome 3 revealed normal disomy pattern. Whole genome Mapping Array demonstrated chromosome gains in 6p, 9q and 17q (Figure 2).

The patient has been followed for three and a half years after brachytherapy with liver function testing and imaging by positron emission tomography-computed tomography (PET-CT) yearly. There has been no evidence of melanoma metastasis.

### Case 2

A previously healthy 59-year-old Vietnamese man presented with sudden onset of decreased vision in the left eye. Best corrected visual acuity was 20/20 in the right eye and Hand Motions at 2 feet in the left eye. The right eye was unremarkable. Funduscopy of the left eye revealed a dense vitreous hemorrhage that obscured details of a choroidal tumor in the superotemporal equatorial region. Ultrasonography showed a dome-shaped choroidal mass with medium to high reflectivity, height of 6.29 mm and basal measurements of 9.36 mm by 11.77 mm.

Following clinical diagnosis of choroidal melanoma, left eye, and systemic medical evaluation that showed no evidence of metastasis, the patient was treated with Iodine-125 brachytherapy. FNAB immediately before plaque application showed densely pigmented cells suspicious for melanoma and consistent with necrotizing melanocytoma. FISH for chromosome 3 revealed normal disomy pattern, but Mapping Array documented chromosome 3 loss and 8q gain (Figure 2).

**Figure 3:**
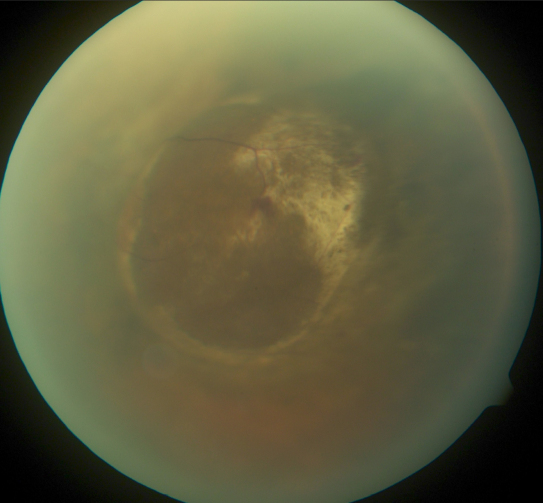
Color fundus photograph of the left eye in Case 2, one year after Iodine-125 plaque brachytherapy, demonstrates a regressed pigmented choroidal melanoma.

One year after brachytherapy, the vitreous hemorrhage had cleared and funduscopy revealed a melanotic choroidal tumor that measured 5.57 mm in height by ultrasonography (Figure 3). Liver function testing and PET-CT imaging was performed yearly. Three years after brachytherapy, imaging studies demonstrated liver lesions that were biopsy-proven to be melanoma metastases.

### Case 3

**Figure 4:**
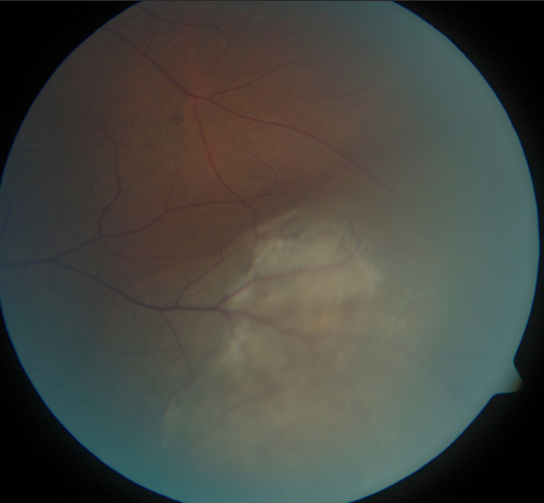
Color fundus photograph of the left eye in Case 3 at initial presentation demonstrates an inferotemporal choroidal melanoma.

A 30-year-old Vietnamese man with a history of laser-assisted in situ keratomileusis (LASIK) two years previously was referred because of pigmented lesion in the left eye detected on LASIK follow-up examination. Visual acuity was 20/20 in each eye. The right eye had no significant abnormality. Funduscopy of the left eye showed a pigmented choroidal tumor in the inferotemporal equatorial region with overlying subretinal fluid (Figure 4). Fluorescein angiography demonstrated intratumor vascularization and ultrasonography revealed a dome-shaped tumor with low internal reflectivity, height of 3.92 mm and basal measurements of 10.70 mm by 11.07 mm.

Choroidal melanoma was diagnosed and systemic medical evaluation showed no evidence of metastasis. The left eye was treated with Iodine-125 brachytherapy. FNAB immediately before plaque placement showed pigmented cells diagnostic of choroidal melanoma and FISH for chromosome 3 had normal disomy pattern. Mapping Array demonstrated chromosomal aberrations consisting of gains in 6p, 8q and 9q as well as losses in 6q and 8p.

The patient has been followed for three and a half years after brachytherapy with liver function testing and imaging by PET-CT yearly. There has been no evidence of melanoma metastasis.

### Molecular analysis

The results of high density, whole genome 250K Mapping Array for the three cases are presented in Figure 2. Loss of one copy of chromosome 3 (monosomy 3) was demonstrated in one patient (Case 2). FISH in this patient showed normal disomy signal pattern, but the patient progressed to biopsy proven liver metastasis three years after brachytherapy.

The two other patients (Case 1 and Case 3) had chromosome 6p gain in the absence of chromosome 3 loss, as well as 9q and 17q gain in Case 1 and gains in 8q and 9q and losses in 6q and 8p in Case 3. These patients have been followed for three years after brachytherapy without evidence of melanoma metastasis.

**Figure 5:**
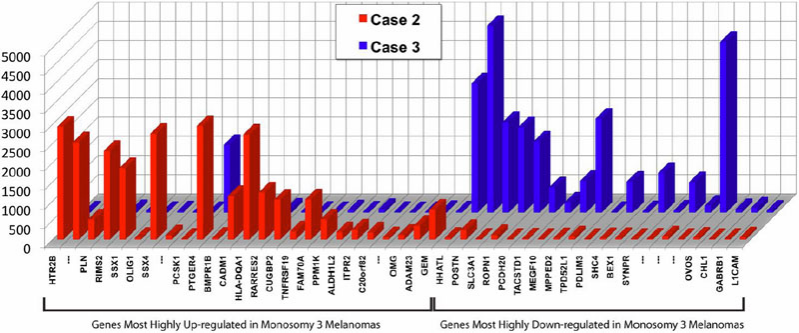
Expression Array Data Comparison of Two Cases. Normalized GeneChip Human U133 Plus 2.0 expression array data comparing Case 2 and Case 3 for mRNA expression in the 25 most highly upregulated genes and 20 most down-regulated genes identified in a previous study of monosomy 3 choroidal melanomas relative to chromosome 6p gain choroidal melanomas [11]. Insufficient RNA was available for analysis of Case 1.

The results of U133 plus 2.0 Array for gene expression for Case 2 and 3 with respect to the 25 most highly upregulated genes and 20 most down-regulated genes identified in a previous study of monosomy 3 choroidal melanomas relative to chromosome 6p [11] choroidal melanomas are shown in Figure 5. Insufficient RNA was available to perform this analysis in Case 1.

## Discussion

In this series of three Vietnamese men, clinical presentation of choroidal melanoma with photopsia, vision loss or discovery on ophthalmologic examination, and appearance of a dome-shaped or mushroom-shaped choroidal mass with subretinal fluid or hemorrhage, and low to medium reflectivity on ultrasonography was comparable to presentation of choroidal melanoma in other ethnic and racial groups. Even the heavy pigmentation in the tumor of Vietnamese men was equivalent to the heavy pigmentation of choroidal melanoma pigmentation reported in Asians from Japan, China, and Thailand [4-6].

Of particular significance in this report of Vietnamese adults were the melanoma cytogenetic abnormalities and gene expression profiles. Cytogenetic aberrations in the choroidal melanomas included chromosome 3 loss by Mapping Array in Case 2 and chromosome 6p gain with normal chromosome 3 disomy in Case 1 and Case 3. Surprisingly, FISH for the centromere of chromosome 3 in Case 2 showed a normal pattern of disomy. This may represent tumor heterogeneity or cells from normal stroma or adjacent normal tissue in the sample studied by FISH. Whole gene Mapping Array, which is a more reliable test^9^, documented chromosome 3 loss in Case 2.

Gene expression in choroidal melanoma of these three Vietnamese men showed profiles of upregulation and down-regulation for the 25 most upregulated and the 20 most down-regulated genes in a comparative gene list. The comparative gene list was derived from a comparison of gene expression in 59 choroidal melanomas comparing melanomas with chromosome 3 loss versus melanomas with 6p gain [11]. Significantly, the Vietnamese choroidal melanoma patient with chromosome 3 loss (Case 2) had a gene expression profile similar to the larger comparative group of choroidal melanomas with chromosome 3 loss. The Vietnamese patient with choroidal melanoma with chromosome 6p gain and the absence of chromosome 3 loss (Case 1) presented a profile of gene expression similar to the larger comparative group of choroidal melanomas with 6p gain.

Overall, clinical presentation of choroidal melanoma in Vietnamese patients closely paralleled the symptoms and signs of choroidal melanoma in other ethnic/racial groups and documented the dense melanin pigmentation characteristic of choroidal melanoma in Asians of Japanese, Chinese, and Thai origin [4-6].

Cytogenetic presentation in Vietnamese patients showed fundamental features of chromosome 3 loss (Case 2) and chromosome 6p gain (Cases 1 and 3) comparable to the cytogenetic characteristics of primarily Caucasian patients. Moreover, the gene expression profile of the patient with chromosome 3 loss (Case 2) and chromosome 6p gain (Case 1) were similar to the gene profiles for the most upregulated and most down-regulated genes in a primarily Caucasian group [11].

Occurrence of biopsy-confirmed melanoma metastasis in the liver of the Vietnamese patient with chromosome 3 loss (Case 2) three years after brachytherapy and no evidence of metastasis in the Vietnamese patients with chromosome 6p gain (Cases 1 and 3) followed for an equal period after brachytherapy was consistent with the high risk of metastasis associated with chromosome 3 loss.

In Case 2, FISH probing for the centromeric region of chromosome 3 yielded disomy 3, which was discrepant with Mapping array data which showed a loss of one copy of chromosome 3. The reasons for this discrepancy may be that tumor heterogeneity resulted from different areas of the tumor biopsied [12-14]. In addition sampling error in the biopsy may have resulted in an analysis of a significant number of macrophages or lymphocytes, rather than actual melanoma cells [15].

Similarities between this small series of three Vietnamese Asians with choroidal melanoma and the larger experience of primarily white patients with choroidal melanoma suggests a similar underlying disease process and infers applicability to the Vietnamese of prognostic information and, ultimately, therapeutic measures derived from larger cohorts of white patients with choroidal melanoma.

Strengths of this report relate to the (1) self-determined identification of Vietnamese Asians on the basis of ancestry and culture, (2) characterization of clinical presentation, diagnosis, treatment and clinical course in Vietnamese men with choroidal melanoma, and (3) use of identical Mapping Array and Expression Array to characterize cytogenetic aberrations and gene expression profiles in Vietnamese men and a larger cohort of white patients with choroidal melanoma. Weaknesses include the (1) small series of three Vietnamese men with choroidal melanoma and (2) rather short post-treatment follow-up of three years.

Although uveal melanoma is rare in Asians, these data indicate that when it does occur, the underlying chromosome and RNA changes are the same as those that occur in Caucasians. In Vietnamese Asians with heavily pigmented choroidal melanoma, clinical presentation, cytogenetic characteristics and gene expression profiles were similar to larger series of primarily white patients with choroidal melanoma. Chromosome 3 loss was associated with a poor prognosis and development of liver metastasis.
